# From De-escalation to Restraint: A Qualitative Study of Emergency Clinician Decision-Making in Patient Agitation

**DOI:** 10.5811/westjem.53798

**Published:** 2026-08-28

**Authors:** Michelle Suh, Beatrice Torres, Elise Brickhouse, Datonye Charles, Ynhi Thomas, Anita Chary

**Affiliations:** * Brown University Health, Department of Emergency Medicine, Providence, Rhode Island; † Brown University, Warren Alpert School of Medicine, Providence, Rhode Island; ‡ Baylor College of Medicine, School of Medicine, Houston, Texas; § University of Washington, Department of Internal Medicine, Seattle, Washington; ¶ Baylor College of Medicine & Michael E. DeBakey VA Medical, Center for Innovations in Quality, Effectiveness and Safety, Houston, Texas; || Baylor College of Medicine, Department of Psychiatry, Houston, Texas; # Baylor College of Medicine, Department of Emergency Medicine, Houston, Texas

## Abstract

**Introduction:**

Managing agitation in the emergency department (ED) is challenging and frequently involves decisions about physical restraints and emergent medications. While institutional protocols and behavioral response teams exist, little is known about how individual clinicians make decisions about restraint use—decisions with significant implications for safety, ethics, and equity. The objective of the study was to explore how emergency physicians, residents, and nurses make decisions about physical restraint and emergent medication use during episodes of patient agitation in the ED.

**Methods:**

We conducted a qualitative study using semi-structured interviews with 35 emergency clinicians (18 attending physicians, 7 senior residents, and 10 nurses) at an urban academic ED. Interviews were transcribed and analyzed using inductive thematic analysis.

**Results:**

Clinicians described three key factors informing restraint decision-making: (1) commitment to patient-centered care through noncoercive de-escalation; (2) real-time assessment of threats to staff and patient safety; and (3) team-based collaboration in both the restraint decision and implementation process. Participants shared a common mental model that prioritized verbal de-escalation before moving to pharmacologic or physical restraint. Clinicians acknowledged that unconscious bias and identity-based dynamics, including age, gender and race, may influence perceptions of threat and decision-making.

**Conclusion:**

Emergency clinicians share a consistent framework for managing patient agitation, suggesting potential for a standardized, team-based algorithm. Future interventions should consider clinician and patient identities, aiming to reduce disparities and enhance safety while minimizing restraint use.

## INTRODUCTION

Acutely agitated patients are common in the emergency department (ED). Studies estimate as many as 1.7 million ED visits in the United States annually involve agitated patients.^1^ The American Association for Emergency Psychiatry’s Project BETA (Best practices in evaluation and treatment of agitation) emphasizes the importance of noncoercive de-escalation as a preferred response.^2^ However, when verbal and behavioral de-escalation techniques are not effective, ED teams may physically restrain and chemically sedate patients to ensure patient and staff safety.

Restraint use in the ED has important consequences and implications for patients, staff, and health equity. First, restraint use is associated with significant patient morbidity, including physical injury and psychological trauma, and potential mortality.^3^–^6^ Second, emergency physicians (EP) and nurses face high rates of workplace violence, specifically from agitated patients.^7^,^8^ Effective de-escalation techniques and restraint use may reduce the risk of violence experienced by ED staff.^9^ Third, recent research has demonstrated that clinicians are more likely to restrain Black and Hispanic patients and men.^10^–^12^ Understanding how clinicians make decisions about restraint use has important implications for addressing workplace violence and health inequities.

Previous research examining restraint use in the ED has largely focused on a systems- and team-level perspective. One qualitative study showed that latent hierarchal structures, power dynamics, and disparate goals of care among team members posed significant challenges to the success of noninvasive de-escalation strategies.^13^ However, interprofessional simulation education regarding agitation events in the ED has been shown to improve communication and cooperation among team members, as well as reinforce dual awareness of both patient and staff safety during agitation events.^14^–^16^ Institutions have created behavioral response teams, typically composed of interdisciplinary staff members, who respond to agitation events in the ED. Studies suggest these behavioral response teams increase staff perceptions of safety and decrease restraint use.^15^,^16^ However, few studies have examined restraint use in the ED from individual clinician perspectives.

One qualitative study of Black, Latino, and multiracial emergency clinicians found perceived bias in restraint use, including staff viewing Black patients as more violent and aggressive and using less de-escalation and engagement prior to applying restraints compared to White patients.^17^ Our qualitative study examining gender and physician experiences with restraint use decision-making showed that female EPs perceived a lack of respect for their clinical authority; they compensated by building consensus among the team to implement their decision to use restraints.^18^ Overall, there is a lack of research at the level of individual emergency clinician decision-making regarding restraint use.

We performed a qualitative study to understand individual emergency clinician perspectives on decision-making about restraint use in an institution that treats acute agitation as a behavioral health code. Understanding clinical decision-making regarding restraint use can provide insights into the individual, team, and systemic factors that inform restraint use and help establish practice standards that minimize restraint use while promoting patient and staff safety.

## METHODS

Our qualitative study focused on emergency clinician decision-making regarding physical restraints and emergent medications in the ED. The Institutional Review Board of Baylor College of Medicine provided ethics approval (H-50511). We used a case study-based approach informed by phenomenology, which elicits the meanings that participants attribute to their experiences—in this case decision-making in the ED. We followed Consolidated Criteria for Reporting Qualitative Research (COREQ) standards for qualitative research (see Appendix A).^19^

**Population Health Research Capsule**

What do we already know about this issue?

*Institutional protocols and behavioral response teams can help de-escalation and agitation management in the emergency department*.

What was the research question?

*We explored individual decision-making of clinicians regarding the use of physical restraint and emergent medication use in agitation*.

What was the major finding of the study?

*Clinicians use a shared mental model of prioritizing de-escalation and assessing threats to patient and staff safety in a team-based approach*.

How does this improve population health?

*A standardized, team-based algorithm could help address disparities in restraint use while enhancing patient and staff safety*.

### Study Setting

The study site was an urban academic ED with an annual visit volume of 80,000 in the Southern United States. The patient population the ED serves has a high level of unmet social needs, with approximately half uninsured and 21% receiving Medicaid.^23^,^24^ Approximately 10% of ED visits are for behavioral health complaints, and the ED has a designated behavioral health patient care area staffed by emergency psychiatrists. Driven by patient outcomes, and regulatory oversight, the institution has increasingly emphasized prioritizing verbal de-escalation and minimizing use of psychoactive medications. In the electronic health record (EHR), orders for violent physical restraints and emergent psychoactive medications must include a justification for use. Restraint use is accompanied by a face-to-face evaluation documented in a note separate from the ED history and physician or nursing notes in the EHR. In 2022, the institution created a crisis intervention team (CIT) composed of an attending EP, emergency psychiatrist, and nursing and security staff, which responds to behavioral health codes—situations in which a patient is agitated and unable to be verbally de-escalated by bedside staff. This behavioral health code is broadcast hospital-wide, comparable to a rapid response code or a code stroke. There are on average 104 behavioral health codes every month.

New staff, including faculty and nurses, undergo orientation on institutional policies for managing agitated patients. All staff must complete institution-specific online training modules, which emphasize institutional criteria for restraint use and proper documentation. However, staff backgrounds and restraint-use training vary significantly. Residents primarily learn on-shift, whereas attendings bring diverse institutional policies, and nursing staff experience high turnover (30% turnover annually since COVID-19). Consequently, prior work experiences drive a high degree of operational variability despite standardized modules.

### Study Sample and Recruitment

Staff nurses, senior residents received recruitment emails via their listserves, while attending EPs received individualized emails to ensure that those recruited to the study worked in the public safety-net hospital, because the other sites served by the academic ED do not have a similar volume of behavioral health patients. Additionally, as described elsewhere,^18^ we sought to have an equal number of women and men respondents to understand how gender might impact experiences regarding application of restraints. Participants received a $50 gift card.

### Data Collection

Led by a senior researcher with qualitative research expertise, the study team developed and pilot tested an interview guide informed by a review of the literature and priorities of the research team (Appendix B). The interview focused on experiences with decision-making regarding ED restraint use, team dynamics, patient factors, and training. Interviews were conducted by video conference; they were digitally recorded and then professionally transcribed.

### Data Analysis

Data analysis was done retrospectively. Using an inductive approach, in which themes emerge from participants’ responses rather than an a priori framework, we reviewed initial transcripts and created a preliminary codebook (Appendix C). Subsequently, each interview transcript was coded independently by two to three team members, with discrepancies in coding resolved through consensus discussion. We elucidated themes through consensus discussions. No new themes emerged after six attending EP interviews, four nursing interviews, and four resident physician interviews, indicating thematic saturation and adequate sample size.^22^ We used a matrix to compare themes that emerged by professional group (attending versus resident physician versus nurse).

The research team included EPs, a psychiatrist, medical students, and public health professionals. We held consensus discussions and ensured that each transcript was coded by both a clinician and a nonclinician to ensure that our themes reflected participants’ responses rather than our own professional experiences.

## RESULTS

### Participant Characteristics

A total of 35 participants completed individual semi-structured interviews. Of those invited to participate, 18 of 41 attendings, 7 of 15 resident physicians, and 10 of 93 nurses completed an interview; the remaining individuals did not respond, Every participant was asked to self-identify their gender, race, and ethnicity. Demographic characteristics are listed in Table 1. Workplace violence was common. Of the total respondents, 67% (12/18) of attendings, 71% (5/7) of senior residents, and 50% (5/10) of nurses had personally been assaulted by a patient in the ED. All 35.participants reported knowing a colleague who had been assaulted.

Three major themes emerged regarding factors influencing individual decision-making on the management of agitated patients: patient-centered strategies for de-escalation; threats to patient and staff safety; and team collaboration (Table 2). Discussion of these factors did not differ by clinical role, although nurses emphasized the threat of workplace violence to themselves and attendings emphasized a feeling of responsibility for the overall team’s safety. Nurses, residents, and attendings held a shared mental model regarding emergent restraint and medication use in the ED.

### Patient-Centered Strategies for De-escalation

Participants placed the patient at the center of decision-making and clinical care. Nurses, residents, and attendings emphasized providing care responsive to patient preferences, needs and values, as well as respecting patient’s rights consistent with the institutional policies. Participants described wanting to minimize harm to the patient and cited the institutional culture of de-escalation, specifically trying various redirection methods before using restraints.

#### First-line choice for management of agitation

Verbal de-escalation and redirection were widely acknowledged to be the first-line choices for management of agitated patients. Nurses and physicians described identifying patient triggers and offering solutions, such as food, blankets, walking around the ED, or a nicotine patch.

[I think through], where are the resources available to help with de-escalation? I make sure I know where things are, like food and water and blankets and physical needs, so that I’m not relying on other people in those situations.

- ID 23, attending

#### Unique patient triggers and de-escalation strategies

Staff also sought to identify triggers and de-escalation strategies specific to each patient.

I had a patient in the waiting room. He was autistic, and he told me he liked [the rubber bubble popping things]. So I ran to the gift shop, bought one, brought it back, and he was very calm. Once we got him out of the waiting room, then we… gave him a computer and let him watch YouTube.

- ID 16, nurse

### Assessing Threats to Patient and Staff Safety

Participants’ perspectives were informed by high rates of workplace violence and a desire to prevent physical harm to both patients and staff. Participants assessed threats to patient and staff safety in the ED based on (1) patient characteristics and (2) prior de-escalation attempts and behavior.

I have had colleagues that got straight up punched, and if whatever happens to that person is debilitating, they have to leave their job. That’s bad for the rest of the patients around them. I think there is a tendency after those events, at least in the acute phase, to…act, rather than wait and see what happens.

- ID 18, attending

#### Patient characteristics are used in threat assessment

Participants commonly cited size, sex, and age as patient factors that affected their assessment of potential threat. Older patients with dementia were viewed as less threatening but higher risk of causing unintentional harm to themselves and staff. In contrast, participants described hypervigilance around younger, larger patients due to higher risk of violence.

If the patient is a 5’1”, 90-pound person, I feel like we can manage any aggression. With a 6’5”, 320- pound person, you get a little nervous for the safety of yourself and safety of your staff. You may be a little bit more aggressive about restraining someone if you think they might get aggressive…. Gender does play a role because men tend to be bigger. There’s a lower threshold to sedate someone who’s a bigger threat to your patient and your staff.

- ID 1, attending

Participants were asked if they had ever been in situations where it seemed like the race of a patient influenced how the care team made decisions about using restraints, given evidence that clinicians disproportionately apply restraints to patients of color. Responses were mixed. Participants often acknowledged that implicit bias exists but denied seeing this in their own practice.

I’ve worked here 18 years, and we’re equal opportunity restrainers: if you are aggressive, or if you are threatening the staff, I feel like you should put staff safety first. I don’t care what color you are.

- ID 13, nurse

However, other participants highlighted patient’s race—specifically Black race—as a key factor influencing their decision-making.

I think that there is a level of fear especially with males of color. When they arrive and they’re loud and aggressive and screaming, there’s probably a lower threshold to restrain and medicate those patients because of not only their race, but also their size and gender.

- ID 12, nurse

I think [clinicians] do tend to get more threatened by Black men. Sometimes…when they talk bigger [it’s] perceived as, oh, he’s getting violent. So, I think sometimes they’re perceived as more of a threat than they actually are.

- ID 15, attending

According to participants, staff from diverse racial and ethnic backgrounds possess specific insights that facilitated the de-escalation of patients with similar backgrounds. The presence of racially diverse staff was cited as a mitigating factor against bias.

Our staff is all races and genders. We do not have a primarily White staff. The nurses, the security guards, and the technicians reflect the patient population who comes in, and they oftentimes are people who I rely on to help with medical decision-making in these domains [of de-escalation]. I think [the bias] is somewhat attenuated because it actually is a diverse population of employees.

- ID 24, attending

#### Prior de-escalation attempts and behavior

Staff attempted to predict a patient’s threat to patient and staff safety based on prior interactions and de-escalation attempts with patients:

I try to factor in what we know about the patient. Is this someone who just came in and we know nothing about? Or is it someone who we’ve seen… throughout their time in the emergency department? If they have been able to be verbally de-escalated in the past, I might be more willing to spend the time to verbally de-escalate.

- ID 5, attending

Participants viewed past violent behavior as an important predictor of threats to safety:

Sometimes patients…have actually made contact with [staff] in some way, whether it was spitting at them or trying to throw something at them…. I’m probably more likely if [the patient] has already been physical with someone to [use sedatives and restraints].

- ID 25, resident

### Team Collaboration

Interviewees described team dynamics as a crucial part of restraint decision-making. Specifically, participants took a team approach to (1) gathering information, (2) communicating and building consensus, and (3) coordinating restraint application.

#### Gathering information

Most nurses, residents, and attendings felt that the ultimate decision to use restraints was made by physicians (80%, 100%, and 83%, respectively). However, participants described the importance of team communication and consensus in the process of deciding whether to use restraints. Both physicians and nurses cited bedside staff, typically nurses and technicians, as having the most information about a patient and their behavior because of their proximity and time spent with the patient. Additionally, bedside staff were seen as more at risk for being assaulted by a patient, particularly if the decision was made not to use restraints.

You always have to consider what the person at the bedside is seeing. I don’t want to wait until one of my staff gets hit before I intervene. There are certainly times where people out of nowhere go off, but in general, we see people more slowly escalate behavior. When you start to see that and people become less redirectable, that’s when you need to intervene.

- ID 28, resident

#### Communicating and building consensus

Physicians described communicating their thought process to ensure everyone on the team had the shared understanding of the situation and next steps.

Once we’ve tried verbally de-escalating, bargaining, [and] a show of force, but the patient is still behaving in [an unsafe way] … You verbalize that out loud. Similarly to what you do in other resuscitations or medical treatment, you say, “Look, this is what I’m seeing. I think we basically have to move down this pathway…meaning chemical sedation and physical restraint.” Then you see if any staff members have objections or other suggestions.

- ID 29, attending

#### Coordination of restraint application

The actual application of emergent restraints and medications was seen as a medically acute situation requiring close coordination of staff. To avoid physical injury to patient or staff, participants valued clear roles, communication, and coordination of staff members. Practices such as a pre-huddle, assigning specific members to designated limbs for restraints, and closed loop communication in the room were cited as ways of ensuring the staff members could work together quickly and efficiently.

We do a team huddle…and gather the care team. We decide who’s tying restraints, who’s holding extremities, who’s going to be watching the airway, who’s going to grab medications, and who’s going to administer medication. It’s very much like a code situation for medical patients. Everyone has a role, everyone has a purpose.

- ID 12, nurse

## DISCUSSION

Our qualitative study found that EPs and nurses prioritized patient-centered de-escalation, valued staff and patient safety, and close team coordination. Clinicians considered patient and team factors and institutional culture and policies when deciding when to use restraints. Our findings support prior research demonstrating that training, as well as explicit behavioral response teams, reinforce dual awareness of staff and patient safety and improve team coordination and cooperation.^13^–^15^ These findings have implications for equipping ED staff with a standardized framework to assess and manage patient agitation as well as decreasing race and sex disparities in restraint use.

There is currently no widely adopted algorithm or pathway used by emergency clinicians to systematically assess and manage patient agitation. Wong et al developed a systems-based workplace violence and agitation care framework with five connected levels: patient; staff; team; ED microsystem; and healthcare macrosystem.^25^ Our findings support and align with Wong et al’s framework, suggesting its applicability across ED settings. However, most institutions rely on a fragmented, institution-specific approach drawing from individual clinician judgment, safety screening tools, clinical pathways, and behavioral response teams.^14^–^16^,^26^–^29^ While these localized approaches may reduce restraint use and improve safety, a standardized and unified clinical pathway could foster a broader shared mental model across emergency medicine practice.

Just as the “Hs and Ts” guide clinicians through reversible etiologies of cardiac arrest, a structured approach to agitation could help ED staff systematically assess threats and tailor responses. Our findings suggest a starting point for such a standardized approach—one that begins with assessing threat to patient and staff, prioritizes noncoercive de-escalation techniques as the first-line response, and emphasizes coordinated team-based action for pharmacologic treatment and restraint use when necessary. Any standardized approach must also account for the role of clinician and patient identities, given evidence that gender and race influence how clinicians navigate leadership and safety decisions during agitation management and that patients gendered as men are more associated with violence.^17^,^18^,^30^,^31^

Our findings also suggest possible mechanisms for the racial and gender disparities surrounding restraint use. First, prior research suggests increased cognitive load may make clinician judgment more susceptible to implicit racial bias.^32^ In our study, participants described trying to ensure their colleagues’ physical safety while also assessing whether a patient’s behavior necessitated restraints. Departmental interventions such as a behavioral response team may decrease the cognitive load for clinicians. Second, staff described assessing threat to patient and staff safety as a key criterion for restraint use. While interviewees felt that patient race did not impact their own decisions, they did acknowledge that bias could impact decisions about restraint use. This suggests interviewees perceived bias in others but not themselves. It is possible that our interviewees were not subject to bias in their patient interactions, that interviewees have implicit bias that they were not aware of, or that interviewees were aware of their own bias but did not want to admit their own culpability. These observations align with psychology research showing that individuals in the United States tend to perceive Black men as threatening,^33^ often overestimating their physical dimensions relative to White men of identical stature.^34^

Dedicated education about implicit racial bias and cultural awareness may be helpful. Developing explicit mechanisms to consider bias has been used in the past, such as the implementation of a “bias check” into decision-making in one emergency medicine residency, where ED staff members were asked to deliberately pause and consider whether a patient’s identity was contributing to perception of threat.^35^ Training staff to explicitly call out racial bias may also be helpful. Third, the use of violence-risk assessment tools may help staff identify patients who may become disruptive. However, many of these risk scores require staff to score factors such as agitation, aggression, and irritability, which are inherently subjective.^28^,^36^,^37^ In fact, research has found that Black patients are more likely to have behavioral flags placed in their chart than White patients, and that Black patients with behavioral flags experience longer ED waiting times and fewer laboratory tests and imaging orders compared to White patients with behavioral flags.^38^,^39^

## LIMITATIONS

Our study was conducted in a single site with a high percentage of behavioral health complaints and institutional features, including a behavioral health code and a designated behavioral health patient care area, which may limit generalizability. The research site was a large urban academic ED, which may have different staff and resources than community, rural, or resource-limited EDs. We did not explore specific populations such as pediatric patients or patients with dementia. Furthermore, our study focused on agitated patients; workplace violence studies have shown ED staff experience significant assaults from visitors and families of patients.^40^,^41^ Additionally, our participants had experienced atypically high rates of workplace violence which may have influenced their decision-making and may limit generalizability.^7^,^42^

Because this was a convenience sample of nurses and physicians who responded to an email invitation, there may have been selection bias in those more interested in this topic, including patient-centered care, equity, safety and threat assessment, and a team-based approach. Additionally, response rates varied substantially across professional groups, particularly among nurses, which may limit the completeness of perspectives captured. As a qualitative study, our findings rely on self-reported clinician perceptions and reflections that are subject to recall bias and social desirability bias, especially when discussing sensitive topics such as race, bias, and restraint use. Lastly, the absence of participant checking (ie, member validation) may limit confirmability of the interpretations.

## CONCLUSION

This study identified that using patient-centered strategies for de-escalation, assessing threats to patient and staff safety, and focusing on team collaboration are important factors informing restraint decision-making in the ED. We also found that staff shared a common mental model prioritizing de-escalation prior to pharmacologic agents or physical restraint. Future work should explore development and implementation of a standardized algorithm, potentially based on the mental model described, to guide responses to patient agitation and clarify mechanisms of racial disparities in restraint use and potential interventions. Future studies should include observation of ED staff behaviors, multicenter validation, and prospective evaluation.

## Supplementary Information







## Figures and Tables

**Table 1:** Demographic characteristics of participants in a qualitative study examining emergency clinician decision-making on restraint use.

Characteristic	Participants (N = 35)
Gender (self-identified)	
Woman	17
Man	18
Nonbinary	0
Race (self-identified)**	
Asian or other Pacific Islander	6
Black or African-American	6
Multiracial	1
White	20
Other	2
Ethnicity (self-identified)**	
Hispanic	9
Non-Hispanic	26
Professional role	
Attending physician	18
Resident physician	7
Nurse	10
Average years of practice (range when applicable)	
Attending physician	10 (4–18)
Resident physician	3
Nurse	9 (1–22)
Prior self-reported de-escalation workplace training (n, %)	
Attending physician	8 (44%)
Resident physician	2 (29%)
Nurse	10 (100%)
Prior restraint workplace training (n, %)	
Attending physician	15 (83%)
Resident physician	4 (57%)
Nurse	10 (100%)

** In cases where participants reported their race as Hispanic, we classified their ethnicity as Hispanic and report race as “Other.” To avoid identifying participants who were underrepresented in emergency medicine, data are provided in aggregate and not by professional role.

**Table 2:** Themes, subthemes, and key quotes in a qualitative study examining emergency clinician decision-making on restraint use.

Category	Subtheme	Key Quotes
Patient-centeredness	First-line choice for management of agitation	“I go through a whole mental checklist before we actually advance to [restraints and medications]. I ask myself is it appropriate to do [restraints and medications]? Have we tried other methods like trying to talk them down, those kind of things?.... Not every patient requires escalation to physical restraints. Some people will do okay if you just talk to them.”
- ID 21, attending		
	Unique patient triggers and de-escalation strategies	“[There was] a patient who just wanted to stand outside his room because he’d been inside the ER for two or three days…. [I would offer] if you want to go walk, we can use the restroom on the other side [of the ED]. We can have security accompany us then just go to the furthest restroom just so you can quote, unquote, get more fresh air.”
- ID 10, nurse		
Assessing threats to patient and staff safety	Patient characteristics	Age definitely plays a factor. The medications we give are not benign medications if and when we need them. So, if someone is older and agitated, you’re less likely to reach for the Haldol or the Versed. You’re more likely to try to physically redirect. And [redirection] does work most of the time.
- ID 27, resident		
	Prior de-escalation attempts and behavior	If it’s a patient that [our staff] have a history with, who has injured someone before or basically assaulted staff, and that’s why they ended up in restraints, then we’re more likely to continue with restraints…because they have already proven their tendency to display violence towards staff.”
- ID 3, attending		
Team collaboration	Gathering information	“Like any other resuscitation, a cardiac arrest/code or anything, you want to take everybody’s input because they might be seeing things that you’re not seeing. And so, I think these type of situations are like psychiatric resuscitation situations.”
- ID 28, resident		
	Communicating and building consensus	“[Sometimes]…the patient’s not a threat. It’s like watching a child who doesn’t want to stay still. They don’t want to relax. They may be harder to direct, but it doesn’t mean that they’re a threat to elope. It doesn’t mean that they’re a threat to somebody’s personal safety. I usually try to reiterate that to the care team like, do you feel unsafe? Or is it that you’re just tired of telling patients to sit down. And most of the time, they’re in agreeance and just deal with it.”
- ID 11, attending		
	Coordination of restraint application	“We have a pre huddle, all the key players and the people who are going to be restraining, the nurses, the doctors. We make sure we come up with a with a plan [like] we’re doing four-point violent restraints. We all agree on that. We’re going to medicate the patient. What medications do we want, [which] nurse is going to go grab the medications. And we delineate roles for how we’re going to handle that situation together.”
- ID 17, nurse		

*ED*, emergency department; *ER*, emergency room.
