# Supplementary material for: From De-escalation to Restraint: A Qualitative Study of Emergency Clinician Decision-Making in Patient Agitation
Source: West J Emerg Med. 2026 Aug 28;27(5):1384–92. doi: 10.5811/westjem.53798 (PMC13660589; doi:10.5811/westjem.53798)
Supplement: Supplementary file 1 [file wjem-27-1384-AppendixC.docx]

| **Code** | **Definition** | **Frequency** |
| --- | --- | --- |
| Patient | Age, frailty, body habitus, sex, drug use, primary language | Attending 100%  Resident 100%  Nurse 100% |
| Institution | Policies about chemical restraints; policies about how/whether people are held; institutional culture or expectations of how to interact with agitated patients | Attending 83%  Resident 86%  Nurse 80% |
| Patient Harm | Concern for imminent physical harm to patient | Attending 94%  Resident 100%  Nurse 100% |
| Staff Harm | Concern for imminent physical harm to staff | Attending 100%  Resident 100%  Nurse 100% |
| Education | Modeling for trainees | Attending 67%  Resident 100%  Nurse 60% |
| Team | Honoring or deferring other team members' perspectives (e.g. of nurse, technician, contract officer/security); recognition that certain team members may agitate or de-escalate a patient | Attending 100%  Resident 100%  Nurse 100% |
| Physician | Gender expectations for the attending, experience of attending | Attending 78%  Resident 100%  Nurse 70% |
| De-escalation | Taking into account prior attempts at de-escalation verbally, or lack thereof | Attending 83%  Resident 100%  Nurse 90% |
| Relationship | Familiarity with patient (clinician has seen multiple times or pt has been under their care for hours versus received signout), rapport, cultural/mutual intelligibility | Attending 78%  Resident 29%  Nurse 70% |
| Proximity | Physical proximity to patient, time at bedside | Attending 78%  Resident 57%  Nurse 60% |
