# Supplementary material for: From De-escalation to Restraint: A Qualitative Study of Emergency Clinician Decision-Making in Patient Agitation
Source: West J Emerg Med. 2026 Aug 28;27(5):1384–92. doi: 10.5811/westjem.53798 (PMC13660589; doi:10.5811/westjem.53798)
Supplement: Supplementary file 2 [file wjem-27-1384-s001.docx]

**COREQ (COnsolidated criteria for REporting Qualitative research) Checklist**

A checklist of items that should be included in reports of qualitative research

| **Topic** | **Item No.** | **Guide Questions/ Description** | **Author Responses** |
| --- | --- | --- | --- |
| **Domain 1: Research team and reflexivity** | | | |
| *Personal Characteristics* | | | |
| Interviewer/facilitator | 1 | Which author/s conducted the interview or focus group? | BT and EB |
| Credentials | 2 | What were the researcher’s credentials? E.g. PhD, MD | Interviewers: Bachelor’s degree  Analysts: Medical students, public health professionals, anthropologist, physicians  Senior researcher: PhD (Anthropology) |
| Occupation | 3 | What was their occupation at the time of the study? | Interviewers: public health and medical students |
| Gender | 4 | Was the researcher male or female? | Female |
| Experience and training | 5 | What experience or training did the researcher have? | Interviewers have previously conducted semi-structured interviews for qualitative studies. They had multiple training sessions with the senior researcher, including practice interview sessions with targeted feedback. |
| *Relationship with participants* | | | |
| Relationship established | 6 | Was a relationship established prior to study commencement? | No |
| Participant knowledge of the interviewer | 7 | What did the participants know about the researcher? e.g. personal goals, reasons for doing the research | Participants knew the interviewer was a medical student, but personal goals and reasons for doing the research were not discussed. |
| Interviewer characteristics | 8 | What characteristics were reported about the inter-viewer/facilitator? e.g. Bias, assumptions, reasons and interests in the research topic | Participants knew the study was examining restraint use in the emergency department, but reasons and interests in the topic were not discussed. |
| **Domain 2: Study design** | | | |
| *Theoretical framework* | | | |
| Methodological orientation and Theory | 9 | What methodological orientation was stated to underpin the study? e.g. grounded theory, discourse analysis, ethnography, phenomenology, content analysis | Case-based informed by a phenomenological approach. Analysis was done using an inductive approach.^1,2^ |
| Sampling | 10 | How were participants selected? e.g. purposive, convenience, consecutive, snowball | The nursing, resident, and attending samples were obtained through volunteer sampling via emails. |
| Method of approach | 11 | How were participants approached? e.g. face-to-face, telephone, mail, email | Email with a reminder email in two weeks if participants had not responded |
| Sample size | 12 | How many participants were in the study? | 35 |
| Non-participation | 13 | How many people refused to participate or dropped out? Reasons? | 8 residents, 83 nurses, and 23 attendings did not participate and did not provide reasons |
| *Setting* | | | |
| Setting of data collection | 14 | Where was the data collected? e.g. home, clinic, workplace | Data were collected via remote videoconference. Participants chose the location, typically their house or office. |
| Presence of non-participants | 15 | Was anyone else present besides the participants and researchers? | No |
| Description of sample | 16 | What are the important characteristics of the sample? e.g. demographic data, date | Participants were all working in an academic emergency department as a nurse, resident, or attending physician. See Table 1 for demographic data. |
| *Data collection* | | | |
| Interview guide | 17 | Were questions, prompts, guides provided by the authors? Was it pilot tested? | The interview guide was created by the research team based on a review of the literature and priorities of the research team, then pilot tested. The interview guide is provided as an Appendix. |
| Repeat interviews | 18 | Were repeat interviews carried out? If yes, how many? | No |
| Audio/visual recording | 19 | Did the research use audio or visual recording to collect the data? | Audio recordings were collected |
| Field notes | 20 | Were field notes made during and/or after the interview or focus group? | There were no field notes |
| Duration | 21 | What was the duration of the interviews or focus group? | Interviews lasted an average of 24 minutes |
| Data saturation | 22 | Was data saturation discussed? | Yes  We used a base of 6 interviews to establish themes. Thematic saturation was reached with 6 attending, 4 nursing, and 4 resident interviews, with no new themes emerging in subsequent interviews.^3^ |
| Transcripts returned | 23 | Were transcripts returned to participants for comment and/or corrections? | No |
| **Domain 3: analysis and findings** | | | |
| *Data analysis* | | | |
| Number of data coders | 24 | How many data coders coded the data? | Six |
| Description of the coding tree | 25 | Did authors provide a description of the coding tree? | Yes (see Appendix C) |
| Derivation of themes | 26 | Were themes identified in advance or derived from the data? | Themes were derived from the data. |
| Software | 27 | What software, if applicable, was used to manage the data? | SaturateApp (saturateapp.com)  Microsoft Excel |
| Participant checking | 28 | Did participants provide feedback on the findings? | No |
| *Reporting* | | | |
| Quotations presented | 29 | Were participant quotations presented to illustrate the themes/findings?  Was each quotation identified? e.g. participant number | Yes |
| Data and findings consistent | 30 | Was there consistency between the data presented and the findings? | Yes |
| Clarity of major themes | 31 | Were major themes clearly presented in the findings? | The three major themes regarding restraint use in the ED are 1) patient-centeredness, 2) threats to patient and staff safety, and 3) team collaboration. |
| Clarity of minor themes | 32 | Is there a description of diverse cases or discussion of minor themes? | We discuss the variable responses to the role of race in decision-making and restraint use. The discussion of physician gender and experiences in leading the team around restraint use are published in a separate manuscript.^4^ |

Developed from: Tong A, Sainsbury P, Craig J. Consolidated criteria for reporting qualitative research (COREQ): a 32-item checklist for interviews and focus groups. *International Journal for Quality in Health Care*. 2007. Volume 19, Number 6: pp. 349 – 357

1.	Bernard HR. Research Methods in Anthropology: Qualitative and Quantitative Approaches, Sixth Edition. Walnut Creek, CA: AltaMira Press; 2002.

2.	Kiger ME, Varpio L. Thematic analysis of qualitative data: AMEE Guide No. 131. Medical Teacher 2020;42(8):846–54.

3.	Guest G, Namey E, Chen M. A simple method to assess and report thematic saturation in qualitative research | PLOS ONE. PLoS ONE [Internet] 2020 [cited 2023 Nov 24];15(5). Available from: https://journals.plos.org/plosone/article?id=10.1371/journal.pone.0232076

4.	Chary A, Torres B, Brickhouse E, Charles D, Thomas Y, Suh M. Gender and emergency physicians’ experiences of leading decision making about restraint use: A qualitative study. Academic Emergency Medicine [Internet] [cited 2024 Apr 23];n/a(n/a). Available from: https://onlinelibrary.wiley.com/doi/abs/10.1111/acem.14851
