# Supplementary material for: From De-escalation to Restraint: A Qualitative Study of Emergency Clinician Decision-Making in Patient Agitation
Source: West J Emerg Med. 2026 Aug 28;27(5):1384–92. doi: 10.5811/westjem.53798 (PMC13660589; doi:10.5811/westjem.53798)
Supplement: Supplementary file 3 [file wjem-27-1384-s002.docx]

**Interview Guide for Physicians:**

Experiences of Decision-Making about Physical and Chemical Restraint Use in the Emergency Department

Thank you for participating in our interview about the use of restraints in the emergency department. Some of the questions can be about difficult topics, so please feel free to take your time to respond or skip a question. Your responses will be confidential and only accessible to the research team. With that being said, do I have permission to record this interview?

Before we get started with the questions, I wanted to state up front that we're interested in your experiences with adult patients, and this interview doesn't concern pediatric patients.

Demographics:

1. How do you identify your gender?
2. How do you identify your race?
3. How do you identify your ethnicity? (Hispanic, non-Hispanic)
4. What is your professional role?
5. How many years have you been practicing emergency medicine?
6. Besides working at Ben Taub, have you worked in other teaching hospitals?
7. Have you worked in other non-teaching hospitals? (yes/no)

| *Question* | *Prompts* |
| --- | --- |
| First off I’ll ask you to think back to the last time you had to decide about using physical or chemical restraints for an agitated patient. Can you tell me about that experience? What was it like? | How did you feel about your own safety?  How did you feel about the safety of other staff members?  How did you feel about what you thought was best for the patient?  How did your sense of the threat level compare to others’ sense of the threat level?  Are there any things you think about specifically when restraining someone? |
| What is your general approach to deciding when to use restraints? When you have an agitated patient, how do you approach making the decision to use physical and chemical restraints?  Does your approach vary on the institution you are working in? | What factors do you take into account when making the decision?  How do you think about your own safety?  The safety of other staff members?  The safety of the patient?  What is best for the patient clinically?  Your sense of the threat level?  Others’ sense of the threat level?  Others’ perceptions of you as a clinical leader?  Institutional policies? |
| When you’re considering using physical or chemical restraints for an agitated patient, how do you take into account concerns of other staff members? | To what extent do you consider other staff members’ perceptions of threat vs. your own?  How do you communicate as a care team about the decision?  How do you approach the situation when you and other team members don’t agree on whether restraints should be used? |
| When you take care of an agitated patient, how do different care team members influence the decision to use physical or chemical restraints?  What influences your decision to use physical or chemical restraints?  Do you feel the final decision ultimately rests with you or another team member? | Are there certain care team members who you think have more influence over the decision than others? If so, who? |
| There’s evidence that clinicians disproportionately apply restraints to men of color.  Have you been in situations where it seemed like the race or sex of a patient influenced how your care team made decisions about using restraints? What have those situations been like?  In your experience, how do other social identities of the patient influence decisions to use restraints? | In your experience, how does an agitated patient's race or sex affect the care team's perception of threat level? |
| Have you or one of your colleagues been physically assaulted by a patient you were taking care of?  How did this effect how you think about de-escalating patients? | What was that experience like? |
| What type of education or training have you had about de-escalating agitated patients? |  |
| What type of education or training have you had about using physical and chemical restraints? |  |

**Interview Guide for Nurses:**

Experiences of Decision-Making about Physical and Chemical Restraint Use in the Emergency Department

Thank you for participating in our interview about the use of restraints in the emergency department. Some of the questions can be about difficult topics, so please feel free to take your time to respond or skip a question. Your responses will be confidential and only accessible to the research team. With that being said, do I have permission to record this interview?

Before we get started with the questions, I wanted to state up front that we're interested in your experiences with adult patients, and this interview doesn't concern pediatric patients.

Demographics:

1. How do you identify your gender?
2. How do you identify your race?
3. How do you identify your ethnicity? (Hispanic, non-Hispanic)
4. What is your professional role?
5. How many years have you been practicing emergency medicine?
6. Besides working at Ben Taub, have you worked in other teaching hospitals?
7. Have you worked in other non-teaching hospitals? (yes/no)

| *Question* | *Prompts* |
| --- | --- |
| First off I’ll ask you to think back to the last time you were on a care team that had to decide about using physical or chemical restraints for an agitated patient. Can you tell me about that experience? What was it like? | How did you feel about your own safety?  How did you feel about the safety of other staff members?  How did you feel about what you thought was best for the patient?  How did your sense of the threat level compare to others’ sense of the threat level?  Are there any things you think about specifically when restraining someone? |
| What is your general approach when your care team is deciding to use restraints? When you have an agitated patient, how do you approach conversations about using physical and chemical restraints?  Does your approach vary on the institution you are working in? | What factors do you take into account when making the decision?  How do you think about your own safety?  The safety of other staff members?  The safety of the patient?  What is best for the patient clinically?  Your sense of the threat level?  Others’ sense of the threat level?  Others’ perceptions of you as a team member?  Institutional policies? |
| When you’re considering using physical or chemical restraints for an agitated patient, how do you take into account concerns of other staff members? | To what extent do you consider other staff members’ perceptions of threat vs. your own?  How do you communicate as a care team about the decision?  How do you approach the situation when you and other team members don’t agree on whether restraints should be used? |
| When you take care of an agitated patient, how do different care team members influence the decision to use physical or chemical restraints?  What influences your decision to use physical or chemical restraints?  Do you feel the final decision ultimately rests with you or another team member? | Are there certain care team members who you think have more influence over the decision than others? If so, who? |
| There’s evidence that clinicians disproportionately apply restraints to men of color.  Have you been in situations where it seemed like the race or sex of a patient influenced how your care team made decisions about using restraints? What have those situations been like?  In your experience, how do other social identities of the patient influence decisions to use restraints? | In your experience, how does an agitated patient's race or sex affect the care team's perception of threat level? |
| Have you or one of your colleagues been physically assaulted by a patient you were taking care of?  How did this effect how you think about de-escalating patients? | What was that experience like? |
| What type of education or training have you had about de-escalating agitated patients? |  |
| What type of education or training have you had about using physical and chemical restraints? |  |
